# Survival, growth and physiology of marine bivalve (*Sinonovacula constricta*) in long-term low-salt culture

**DOI:** 10.1038/s41598-019-39205-2

**Published:** 2019-02-26

**Authors:** Peng Maoxiao, Liu Xiaojun, Niu Donghong, Ye Bo, Lan Tianyi, Dong Zhiguo, Li Jiale

**Affiliations:** 1 Key Laboratory of Exploration and Utilization of Aquatic Genetic Resources and College of Fisheries and Life Science, Shanghai Ocean University, Shanghai, 201306 China; 2 Co-Innovation Center of Jiangsu Marine Bio-industry Technology, Huaihai Institute of Technology, Lianyungang, 222005 China

## Abstract

In this study, we investigated the possibility of rearing and breeding the razor clam (*Sinonovacula constricta*) in inland low salinity water or freshwater. Long-term low salinity (LS) rearing was performed for 3 months to determine the effects of LS on the survival rate, growth rate, and the activities of critical enzymes in juvenile *S. constricta* (JSC). The survival rate in the LS group was only 15.67% at the end of the LS rearing test. In the first month, the survival rate in the LS group was significantly lower than that in the control group (*P* < 0.001). The growth rate (shell length growth rate and weight gain rate) was significantly lower in the LS group than the control group in the first month (*P* < 0.001 for length and weight). However, the growth rates in the two groups differed little during the second and third months. The oxygen consumption and ammonia excretion rates by JSCs were significantly higher in the LS group than the control group during the first month, but they decreased gradually during the following 2 months. The Na^+^/K^+^ ATPase and superoxide dismutase activities were significantly higher in the LS group than the control group during the first month, but they then decreased gradually until there were no significant differences between the groups. However, the aspartate aminotransferase activity was higher in the LS group than the control group during all 3 months. Most of the JSCs died due to LS but the survival of some JSCs suggests the possibility of breeding LS resistant or freshwater *S. constricta*.

## Introduction

The use of inland low salinity water (ILSW) to culture marine fish and shrimps is effective for increasing aquaculture production^1–3^. The presence of saline soils in more than 100 countries leads to surface water and groundwater with a salinity of more than 1 ppt^4^. The Pacific white shrimp, *Litopenaeus vannamei*, is considered to be the most successful invertebrate cultured in low salt conditions. Due to the success of *L. vannamei* aquaculture in Thailand using a low salinity water containing carbonate, then the culture mode of ILSW, Inland saline water and freshwater were practiced throughout the world^5^. The production of *L. vannamei* exceeds 70% of the total global shrimp production^5^. *L. vannamei* can be farmed in ILSW with low salinity but also in freshwater with a salinity less than 0.5 ppt^6,7^. *L. vannamei* is considered to be a strong regulator of osmotic pressure and it can tolerate a wide range of salinities (0.5–45 ppt)^8,9^. In addition, other shrimp have been tested to determine the possibility of their cultivation in ILSW, such as *Penaeus latisulcatus*^10^ and *Penaeus monodon*^11–13^.

Many studies have investigated the acute tolerance of low salinity (LS) by shellfish^14–16^ based on explorations of the range of acute low salt tolerance by shellfish species and the mortality caused by reduced salinity during the breeding process. However, few long-term LS acclimation studies have been reported with the aim of shellfish culture in ILSW and freshwater. Only six kinds of shellfish have been cultivated in inland saline alkaline waters with high salinity levels, i.e., *Cyclina sinensis*^17^, *Mytilus edulis*^18^, *Haliotis laevigata*^19^, *Trochus niloticus*^20^, *Crassostrea gigas*, and *Saccostrea glomerata*^12^, However, no marine shellfish have been cultured in ILSW (very LS) or freshwater, except for *L. vannamei*.

Pre-culture acclimation to the environment is critical for the LS culture of aquaculture animals. At present, ILSW aquaculture and freshwater aquaculture with marine fish and shrimps require gradient acclimation with seedlings^21–23^. A single gradient can be used for acclimation to reduced salinity or multiple gradients to slowly acclimate to reduced salinity, which has a great impact on the survival rate of seedlings^24^. Multiple gradients with slow reductions in salinity can facilitate acclimation and lead to higher seedling survival rates. This acclimation method can also allow animals to adapt better to low salt conditions^25^.

The most important effect of ILSW and freshwater under excessively low salinity is on the osmotic pressure in marine animals^26^. Clearly, problems will occur due to imbalances in the proportions of ions, but these issues are not discussed further in this study. The effects of low osmotic pressure in the environment are great and they are thought to directly affect the survival, growth, metabolic levels, and immune responses in fish and shrimp^27,28^. Robust osmoregulators can avoid these effects and better breeding results may be obtained. In addition, acclimation can reduce these effects^26^.

*Sinonovacula constricta* is the one of the most important edible bivalve species in China, and it is distributed in the western Pacific Ocean^29^. *S. constricta* is a species that tolerates a wide range of temperatures and acute salinity from 1.8–40 ppt^16^. Many studies have investigated the effects of water quality parameters on the physiology and growth of *S. constricta*, such as salinity^30^, metal ions^31^, inorganic pollutants^32^, organic pollutants^33^, pH^34^ and carbonate alkalinity^35^. The effects of salinity on survival and metamorphosis by *S. constricta* larvae have also been investigated^16^. However, studies of low salinity acclimation and long-term low salinity aquaculture using *S. constricta* juveniles with the aim of ILSW and freshwater culture have not been reported.

*S. constricta* exhibits good environmental tolerance and it requires further investigation because the current saline and freshwater fisheries in China lack edible shellfish farming on an economic scale^36^. Shellfish aquaculture is considered to be an ecologically sustainable activity^37–39^. In addition, shellfish consume algae in the water to keep the ecological health^40,41^. Therefore, we investigated the long-term survival and physical activity of juveniles *S. constricta* (JSC) under long-term LS stress in order to determine the possibility of using *S. constricta* as a breeding species in ILSW or freshwater in China.

## Materials and Methods

The animals in this experiment were treated according to the guidelines for the care and use of experimental animals set by the Institutional Animal Care and Use Committee of Shanghai Ocean University (IACUC-SHOU), Shanghai, China. The examination and approval number SHOU-DW-2018-012. The test animals were non-endangered animals and were artificially propagated larvae.

30-days-old healthy JSCs were collected from Donghang Farm, Sanmen City, Zhejiang Province, China. 100 JSCs were used to measure their initial data (average body weight 0.0164 ± 0.0027 g, average shell length 0.6184 ± 0.069 cm). Sea salts (Red Sea, Red Sea Fish Pharm Ltd, Israel) was used to produce the artificial seawater (ASW). Beach mud collected from the East China Sea at Lingang New City, Shanghai City, China. The test beach mud was produced as described previously^35^. During the test, the water temperature was controlled at 20–22 °C.

### Long-term LS stress test

Before test JSCs were acclimated in 10 ppt ASW for 10 days. Two test groups were set up for the long-term stress tests comprising control and LS groups and every group sets three separate tests. Every separate test was performed in a tank (40 × 40 × 65 cm). Each tank contained JSCs (100 randomly selected), test beach mud (10.4 L) and test water (12 L). 8 days gradual acclimation was performed In LS group (Table 1). The final test water conditions of control and LS groups were show in Table 1 (D8). In addition, the measure value of *C*_A_ in control and LS groups were 1.22 ± 0.08 mmol · L^−1^ and 0.28 ± 0.02 mmol · L^−1^, respectively. Daily changing test water and feeding method as described previously^35^. During the 100-day stress test (sampling was analyzed on day 8, day 39, day 69, and day 100), we counted the surviving JSCs as well as measuring the growth rate, oxygen consumption rate, ammonia excretion rate, and enzyme activities.

**Table 1:** Design of the gradual acclimation scheme for the long-term LS stress test.

Acclimation time (days)	Control			LS		
	Setting salinity (ppt)	Setting *C*_A_ (mmol · L^−1^)	Setting pH	Setting salinity (ppt)	Setting *C*_A_ (mmol · L^−1^)	Setting pH
D1	6	0	8.2	6	0	8.2
D2	6	0	8.2	5	0	8.2
D3	6	0	8.2	4	0	8.2
D4	6	0	8.2	3	0	8.2
D5	6	0	8.2	2	0	8.2
D6	6	0	8.2	1.5	0	8.2
D7	6	0	8.2	1	0	8.2
D8	6	0	8.2	0.5	0	8.2

### Oxygen consumption rate and ammonia excretion rate

JSCs were placed in a sealed conical flask to measure the oxygen consumption and ammonia excretion^42^. The oxygen consumption and ammonia excretion rates of 0-day were measured using 60 randomly selected JSCs. For the control, we tested 60 JSCs (three separate tests with 20 JSCs in each group) per month. There were few individuals in the LS group so all of the surviving JSCs were tested. Dissolved oxygen contents was determined using a dissolved oxygen analyzer (WTW Multi 3420 Set G, Xylem Inc., Germany). Ammonia nitrogen was determined using Nessler’s reagent colorimetric method^43^.

### Enzyme activities

The enzyme activities of 0-day were measured in nine randomly selected JSCs. Nine JSCs (three separate tests with three JSCs in each group) when then tested each month in the control and LS groups used. Using normal saline to dilute homogenized tissue samples (After shelled JSC) at a ratio of 1 g:9 ml (tissue weight: normal saline). The diluted samples were then used to determine the activities of Na^+^/K^+^ ATPase (NKA), aspartate aminotransferase (AST) and superoxide dismutase (SOD). Coomassie Brilliant Blue Total Protein Assay kit (Nanjing Jiancheng Bioengineering Institute, China) was used to determine the total protein contents in the diluted tissue samples^44^. Ultra Trace Na^+^/K^+^ ATPase Assay kit (Nanjing Jiancheng Bioengineering Institute, China), Aspartate Aminotransferase Assay kit (Nanjing Jiancheng Bioengineering Institute, China) and Superoxide Dismutase Assay kit (Nanjing Jiancheng Bioengineering Institute, China) were used to measure the activities of NKA (units (U) as μmol · Pi · mg · protein^−1^ · h^−1^), AST (units (U) as μmol · mg · protein^−1^ · min^−1^) and SOD, respectively, according to the manufacturer’s instructions. The units (U) of SOD as the enzyme content of 50% SOD inhibition in the reaction system.

### Calculation and data Analytical methods

Per month survival rate (PMSR)

$${\rm{PMSR}}( \% )=100\ast {N}_{e}/{N}_{(e\mbox{--}1)}$$

*N*_*(e* − *1*)_ and *N*_*e*_ are the numbers of surviving individuals in the (e − 1)-th and e-th month, respectively.

Weight gain rate (WG)

$${\rm{WG}}( \% \,{{\rm{day}}}^{\mbox{--}1})=100\ast ({W}_{e}\mbox{--}{W}_{(e\mbox{--}1)})/T$$

*W*_*(e* − *1)*_ and *W*_*e*_ are the wet body weights of the individuals in the (e − 1)-th and e-th month, respectively. *T* is a months (30 days).

Shell length growth rate (SGR)

$${\rm{SGR}}( \% \,{{\rm{day}}}^{\mbox{--}1})=100\ast ({L}_{e}\mbox{--}{L}_{(e\mbox{--}1)})/T,$$

*L*_*(e* − *1)*_ and *L*_*e*_ are the lengths of the individuals in the (e − 1)-th and e-th month, respectively. *T* is a months (30 days).

Oxygen consumption rate

$$\begin{array}{rcl}({\rm{mg}}\cdot {{\rm{O}}}_{{\rm{2}}}\cdot {{\rm{g}}}^{\mbox{--}1}\cdot {{\rm{L}}}^{\mbox{--}1}\cdot {{\rm{h}}}^{\mbox{--}1}) & = & [{A}_{i}(\mathrm{initial}\,{\rm{oxygen}}\,\mathrm{concentrations})\\  &  & \mbox{--}\,{A}_{f}({\rm{final}}\,{\rm{and}}\,{\rm{initial}}\,{\rm{oxygen}}\,{\rm{concentrations}})]\\  &  & \ast \,V({\rm{volume}}\,{\rm{of}}\,{\rm{test}}\,{\rm{water}})\\  &  & /[W({\rm{wet}}\,{\rm{body}}\,{\rm{weight}})\ast T({\rm{test}}\,{\rm{time}})]\end{array}$$

Ammonia excretory rate

$$\begin{array}{rcl}({\rm{mg}}\cdot {{\rm{g}}}^{\mbox{--}1}\cdot {{\rm{h}}}^{\mbox{--}1}) & = & E({\rm{ammonia}}-{\rm{N}}\,{\rm{content}})\\  &  & /[W({\rm{wet}}\,{\rm{body}}\,{\rm{weight}})\ast T({\rm{test}}\,{\rm{time}})]\end{array}$$

### Statistical analysis

SPSS 19.0 statistical software was used to perform statistical analyses. Significant differences among treatments were determined using one-way ANOVA analysis with Tukey’s test. Sigmaplot 12.3 software was used to plot figures.

### Ethical approval

All applicable international, national, and/or institutional guidelines for the care and use of animals were followed.

## Results

### JSC survival under long-term LS stress

**Figure 1:**
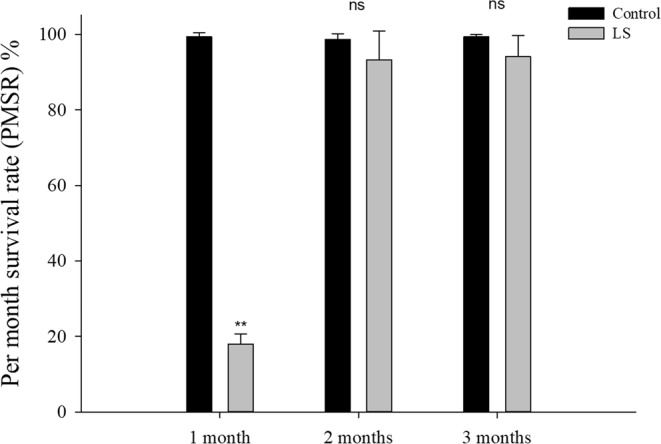
JSC survival rates per month in the long-term LS stress test. Bars (mean ± SE, n = 3) with an asterisk denote a significant difference (^*^P < 0.05, ^**^P < 0.001, ns: no significant difference) between the two groups in each month.

The numbers of JSC deaths each month during long-term LS stress are shown in Table 2. The survival rate of JSCs after 100 days was significantly higher (*P* < 0.001) in the control group (97.33 ± 1.528%) than the LS group (15.67 ± 1.528%). Figure 1 shows that PMSR differed in the two groups after first month, where PMSR was significantly lower in the LS group than the control group (*P* < 0.001). In second and third month, high survival rates were maintained in the control group and LS group and there were no significant differences between them.

**Table 2:** Growth and death of JSC in the two groups in the long-term LS stress test.

Measurement items	Groups	Culture time		
		1 month	2 month	3 month
Shell length (cm)	Control	0.968 ± 0.141	1.098 ± 0.075	1.268 ± 0.120
	LS	0.723 ± 0.012^**^	0.842 ± 0.017^**^	1.109 ± 0.024^*^
Body weight (g)	Control	0.062 ± 0.004	0.102 ± 0.009	0.158 ± 0.005
	LS	0.024 ± 0.001^**^	0.046 ± 0.002^**^	0.102 ± 0.005^**^
The number of deaths	Control	0.667 ± 1.155	1.333 ± 1.528	0.667 ± 0.577
	LS	82.000 ± 2.646^**^	1.333 ± 1.528 ^ns^	1.000 ± 1.000 ^ns^

Values (expressed as mean ± SE) with an asterisk denote a significant difference (^*^P < 0.05, ^**^P < 0.001 ns: no significant difference) between the two groups for the same index in each month.

### JSC growth under long-term LS stress

**Figure 2:**
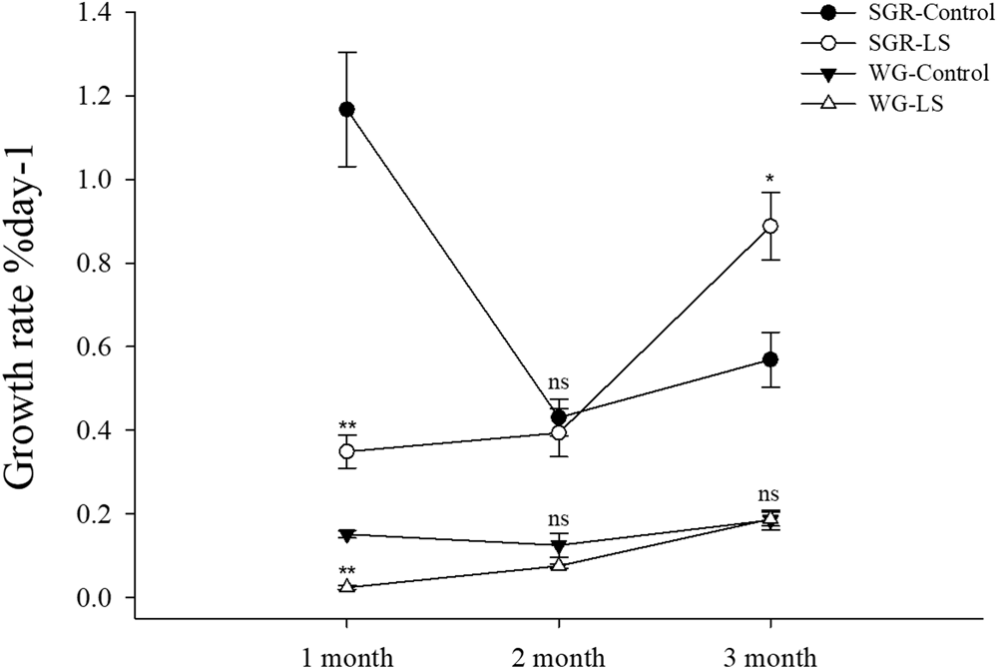
JSC growth rate in the long-term LS stress test. Bars (mean ± SE, n = 3) with an asterisk denote a significant difference (^*^P < 0.05, ^**^P < 0.001, ns: no significant difference) between the two groups in each month.

Direct measurements obtained for the shell length and body weight during the three months are shown in Table 2. The JSCs continued to grow during each month in both groups. The shell length and body weight were significantly lower in the LS group than the control group (*P* < 0.05) throughout the 100-day test period. Figure 2 show that SGR and WG were significantly lower in the LS group than the control group (*P* < 0.001) during the first month. The SGR was no significant differences in the second month, and it was significant higher in the LS groups than the control group (*P* < 0.05) in the third month. The WG was significant lower in the LS groups than the control group (*P* < 0.05) in the second month, and it was no significant differences in the third month.

### Oxygen consumption and ammonia excretion by JSCs under long-term LS stress

**Figure 3:**
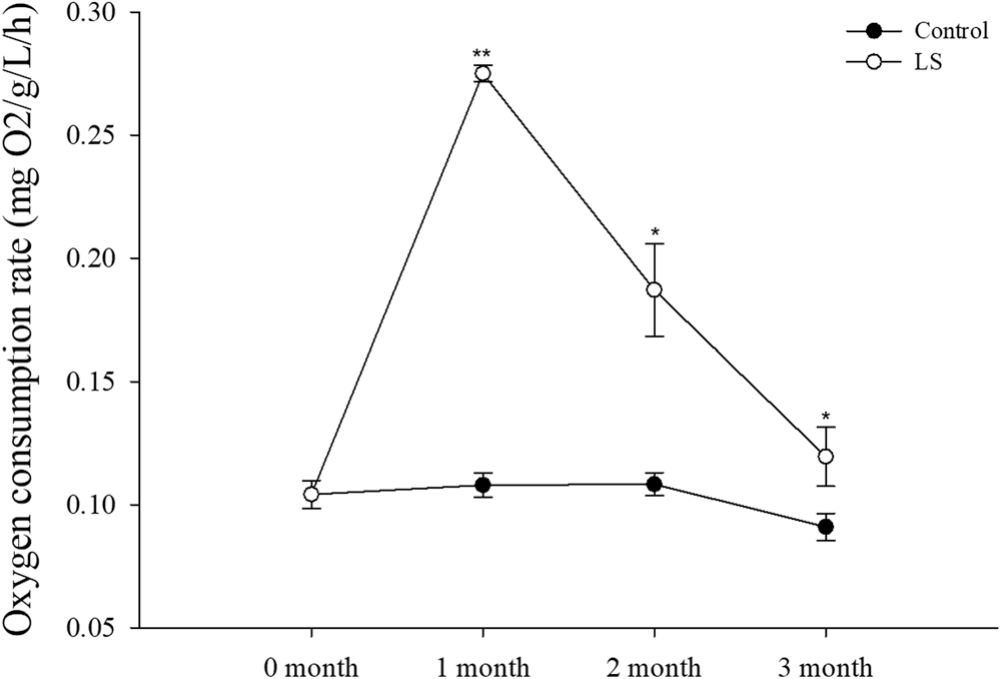
JSC oxygen consumption rate in the long-term LS stress test. Bars (mean ± SE, n = 3) with an asterisk denote a significant difference (^*^P < 0.05, ^**^P < 0.001, ns: no significant difference) between the two groups in each month.

**Figure 4:**
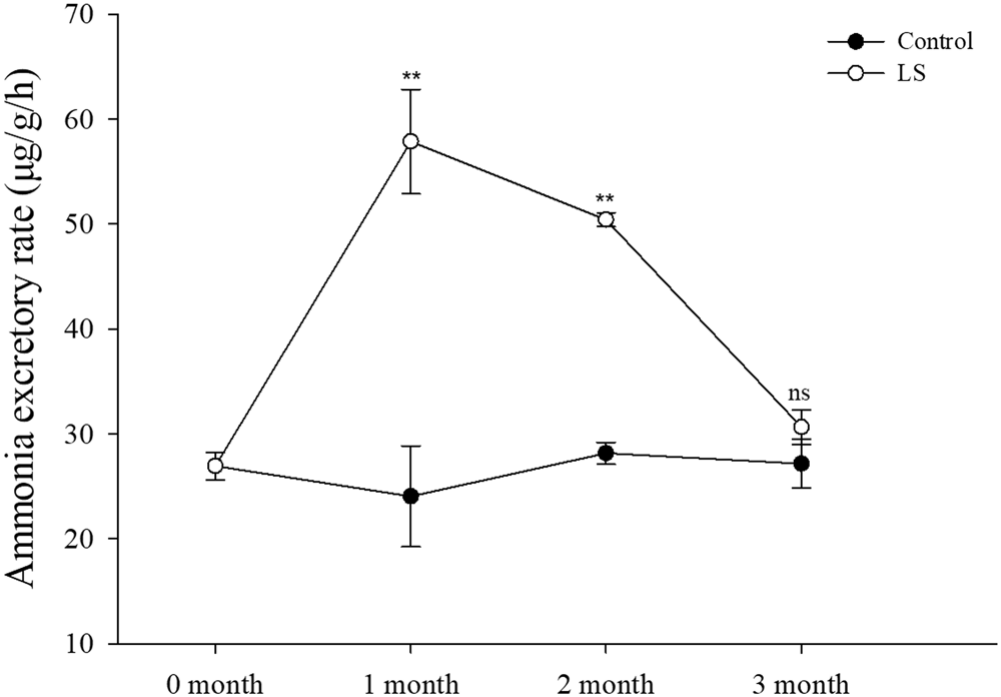
JSC ammonia excretion rate in the long-term LS stress test. For the convenience of display the unit of ammonia excretory rate was converted to (μg · g^−1^ · h^−1^). Bars (mean ± SE, n = 3) with an asterisk denote a significant difference (^*^P < 0.05, ^**^P < 0.001, ns: no significant difference) between the two groups in each month.

Figure 3 show that the oxygen consumption rate was significantly higher in the LS group than the control group during each month. The results in Fig. 4 are similar to those in Fig. 3 but in the third month show that the ammonia excretion rates in the LS group did not differ significantly from those in the control group.

### Enzyme activities under long-term LS stress

**Figure 5:**
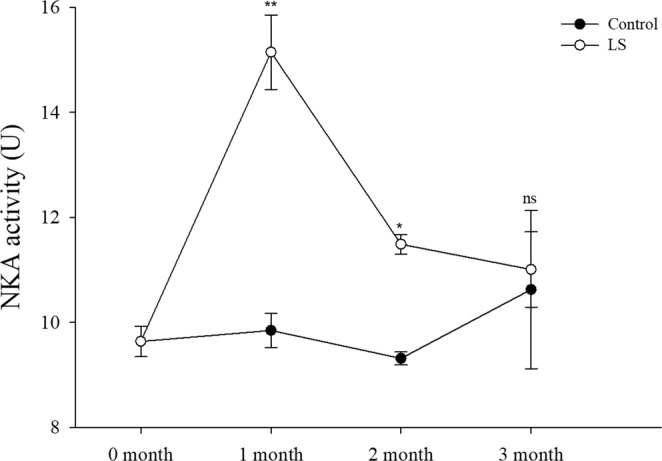
JSC NKA activity levels in the long-term LS stress test. Bars (mean ± SE, n = 3) with an asterisk denote a significant difference (^*^P < 0.05, ^**^P < 0.001, ns: no significant difference) between the two groups in each month.

**Figure 6:**
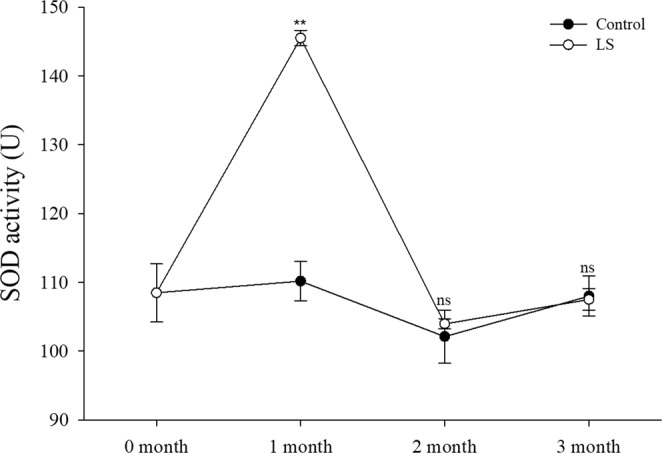
JSC SOD activity levels in the long-term LS stress test. Bars (mean ± SE, n = 3) with an asterisk denote a significant difference (^*^P < 0.05, ^**^P < 0.001, ns: no significant difference) between the two groups in each month.

The NKA activity (Fig. 5) and SOD activity (Fig. 6) were significantly higher in the LS group than the control group during the first month (*P* < 0.001 for NKA activity, *P* < 0.001 for SOD activity), but they did not differ significantly in the third months. In the second month NKA activity was significantly higher in the LS group than the control group, but SOD activity did not differ significantly from those in the control group.

**Figure 7:**
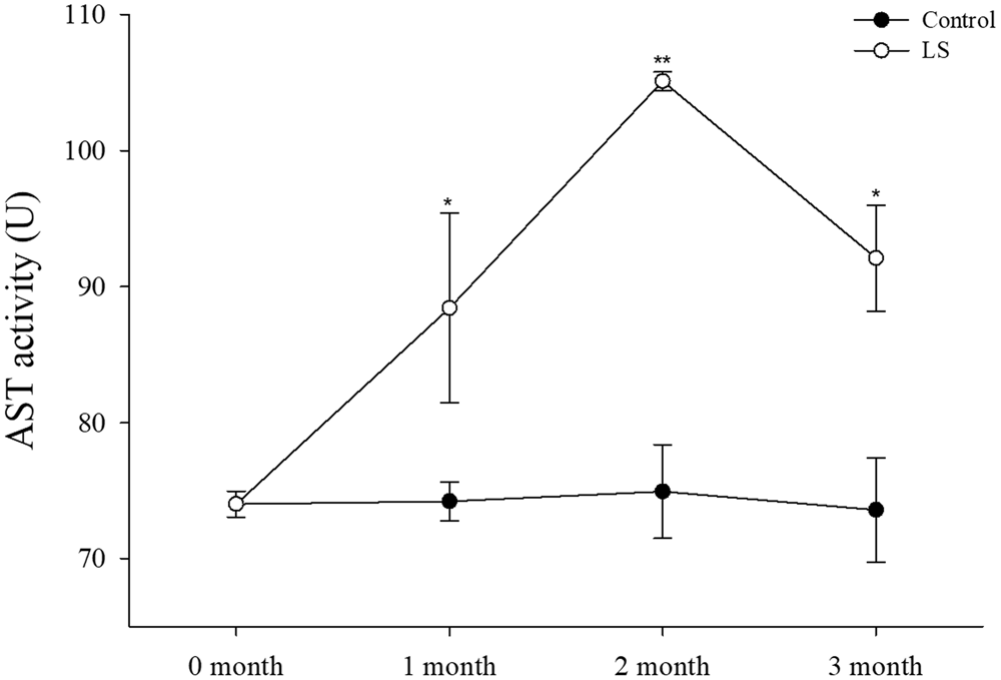
JSC AST activity levels in the long-term LS stress test. Bars (mean ± SE, n = 3) with an asterisk denote a significant difference (^*^P < 0.05, ^**^P < 0.001, ns: no significant difference) between the two groups in each month.

Figure 7 shows that the AST activity was significantly higher in the LS group than the control group during each month (month 1, *P* < 0.05; month 2, *P* < 0.001; month 3, *P* < 0.05).

## Discussion

*S. constricta* as an estuarine intertidal zone bivalve and it naturally experiences many water quality changes. We have studied the growth traits of *S. constricta* for many years in our laboratory. Our findings and previous studies suggested that *S. constricta* is potentially a shellfish with a strong capacity for adapting to different environments. The possible adaptability of *S. constricta* can be considered in the following context. In its original living environment, Qi^45^ found that when the salinity was 26 ppt or less, the survival rate of *Scylla paramamosain* larvae in the Chinese Hainan population was significantly higher than that of the Chinese North Sea population and the Vietnamese population. This was explained by the parents of the Hainan population living in a LS environment (13–20 ppt). Lin and Wu^30^ also suggested that *S. constricta* larvae with parents that live in lower salinity environments can adapt better to LS environments. According to a previous study of *L. vannamei*, differences in the growth stages have important effects on individual LS tolerance. Jayasankar *et al*.^46^ showed that the survival rates increased with the postlarval age in *L. vannamei* but declined as the salinity decreased. Juveniles could tolerate much lower salinities than postlarval individuals. The salinity requirements are different for *S. constricta* larvae and adults, where the larvae often require lower salinity. In order to obtain a more general understanding of LS aquaculture with *S. constricta*, juveniles bred from *S. constricta* parents that lived in normal salinity conditions (12–25 ppt) were selected for testing in the present study. In addition, low salinity acclimation is required before cultivation because it can enhance the survival of LS cultured animals. Kumlu *et al*.^24^ found that the transfer of *Penaeus indicus* postlarvae directly from 30 ppt salinity to 5 ppt without acclimation resulted in a large number of individual deaths. Jayasankar *et al*.^46^ and McGraw *et al*.^25^ studied the effects of different acclimation methods on the survival rate in *L. vannamei*, and found that prolonging the acclimation time was more favorable for reducing the stress due to the environmental osmotic pressure and a higher survival rate was obtained. Based on the results of preliminary experiments and a previous study of *L. vannamei*^25^, we selected an acclimation period of 8 days for JSCs at salinity levels from 6 ppt to 0.5 ppt. The results showed that the acclimation scheme in Table 1 achieved a high survival rate and greater vigor during acclimation.

According to the survival rate results (Table 2 and Fig. 1), LS had the greatest inhibitory effect on the survival of JSCs at the end of the first month, before the mortality rate stabilized and few deaths occurred subsequently in the control and LS groups. The same trend was reflected by the shell length growth and weight gain (Fig. 2) results. At the end of the second month, the SGR and WG values were show different between the LS group and the control group, thereby suggesting that the adaptation of JSCs to LS may require a longer time and that JSCs may differ in energy dominance under low-salt stress. Laramore *et al*.^47^ found that at the end of one month, growth by *L. vannamei* was significantly lower at 2 ppt salinity than that at 30 ppt salinity. JSCs could adapt well to LS environments in long-term culture but there were individual differences in adaptability. Thus, more than 80% of the JSCs died by the end of the first month and the final survival rate was only 15.67% at the end of the third month. Jayasankar *et al*.^46^ found that the survival rate of *L. vannamei* in water with 1.5 ppt salinity was only 45% after 12 weeks of culture. Therefore, individual differences may exist, thereby indicating that the possibility of selective LS breeding with *S. constricta* to obtain LS-tolerant *S. constricta* varieties. To survive in a LS environment, the JSCs needed to consume more energy in order to maintain their physiological needs, which affected energy accumulation, thereby leading to slower growth and even death^48,49^. Figures 3 and 4 show that the oxygen consumption and ammonia excretion rates increased significantly in the JSCs under LS culture, but they decreased gradually over time. Thus, the higher metabolism of the JSCs in the LS group may have led to a growth lag and high mortality. Fan *et al*.^42^ reported that the assimilation rate in *S. constricta* adults treated acutely with 6 ppt salinity was significantly lower than that under 22 ppt salinity. Emerson^50^ and Haberfeil *et al*.^51^ also showed that the metabolism increased in aquatic invertebrates as the salinity decreased. Pressley *et al*.^52^ noted that living in a LS environment can cause the loss of sodium ions. In order to compensate for this loss, it is necessary to activate sodium ion absorption processes. Thus, the increased oxygen consumption and ammonia excretion rate may have been related to the absorption of sodium ions.

Clearly, the physiological changes were related, where LS first caused changes in the osmotic pressure. The body permeability decreases when broad salt marine crustaceans are exposed to a LS environment, where water excretion increases and organic osmolytes in the body fluids are regulated to decrease the osmotic pressure difference between the environment and the hemolymph^26^. Osmotic pressure regulation is a complex physiological process, but sodium and potassium regulation are mainly mediated by NKA on the gill cells^5^. The NKA activity was significantly higher in the LS group than the control group in the first month according to Fig. 5, and similar results have been reported previously^53–56^. The NKA activity was obviously affected by osmoregulation and this consumed large amounts of energy in the JSCs. Huai *et al*.^57^ and Jin *et al*.^58^ consider that changes in the AST levels are consistent with the growth rate in *L. vannamei*. However, in the present study, we found that the AST activity increased significantly only in the first month in the LS group (Fig. 7), which may have been related to the role of AST in response to assimilation^59^. In order to response with changes in salinity in the environment, aquatic organisms passively lose water or absorb water, causing changes in osmotic pressure, NKA enzymes are activated which promote changes in the body’s metabolism until the osmotic pressure tends to stabilize^60,61^. Thus, the increased NKA activity (osmotic pressure changes) may have increased the oxygen consumption rate, ammonia excretion rate, and AST activity in the first month. The effect of osmotic pressure was crucial and it led to decreased survival and growth. The regulation of salinity changes in crustaceans such as *Callinectes danae*^62^, *Portunus trituberculatus*^63^ and *L. vannamei*^64^ mainly includes passive stress period, active regulation period and adaptation period. In the test of low salt stress (15 ppt) of *Anadara broughtonii* juvenile shellfish, NKA enzyme activity gradually stabilized after undergoing peak change, and NKA enzyme activity in low-salt group was higher than that of control at stress to 168 h^61^. In the study of *C. sinensis* (seawater shellfish), the NKA activity was significantly increased after an acute decrease in salinity (23 ppt → 16 ppt), and reached a peak on the 18th day and then fell back slightly to the initial level^65^. It suggest that the NKA activity decreased in the second and third months when the effect of osmotic pressure decreased, and thus growth began to accelerate with greater oxygen consumption, and the ammonia excretion rate and AST activity were maintained at relatively high levels. Li *et al*.^2^ found that exposing *L. vannamei* to LS water for a short time significantly increased the SOD activity level. However, Lin *et al*.^66^ found no differences in the SOD activity levels in a 2.5 ppt group and 5 ppt group at the end of culture for 24 weeks with *L. vannamei*. Dong *et al*. found that the activity of SOD in the *Apostichopus japonicus* at 16 ppt increased first and then decreased, which was thought to be related to the excessive oxygen stress generated by the metabolism under low salt stress^67^. It means that SOD may be needed to scavenge the free radicals produced by metabolism during the maintenance of aquatic animal health^68^.

## Conclusions

A decrease in the environmental osmotic pressure can lead to the loss of sodium ions from the body and an increase in the NKA activity. This increase in NKA activity leads to increased metabolic consumption and ammonia excretion, with increased oxygen consumption, AST activity levels, and metabolic free radicals, thereby leading to higher SOD activity levels. The several JSCs that could tolerate the combined effects of these physiological process and have survived, but the energy required led to reduced growth rates. However, some of the JSCs could adapt to these effects under long-term stress and these individuals diverted more energy to growth. Therefore, *S. constricta* has potential as a species for breeding in ILSW or freshwater in China.
